# Crystal structure control of aluminized clay minerals on the mobility of caesium in contaminated soil environments

**DOI:** 10.1038/srep43187

**Published:** 2017-02-24

**Authors:** Liva Dzene, Eric Ferrage, Jean-Christophe Viennet, Emmanuel Tertre, Fabien Hubert

**Affiliations:** 1 Université de Poitiers/CNRS, UMR 7285 IC2MP, Equipe HydrASA, 5 rue Albert Turpain, Bât. B8, TSA - 51106, 86073 Poitiers cedex 9, France

## Abstract

Radioactive caesium pollution resulting from Fukushima Dai-ichi and Chernobyl nuclear plant accidents involves strong interactions between Cs^+^ and clays, especially vermiculite-type minerals. In acidic soil environments, such as in Fukushima area, vermiculite is subjected to weathering processes, resulting in aluminization. The crystal structure of aluminized clays and its implications for Cs^+^ mobility in soils remain poorly understood due to the mixture of these minerals with other clays and organic matter. We performed acidic weathering of a vermiculite to mimic the aluminization process in soils. Combination of structure analysis and Cs^+^ extractability measurements show that the increase of aluminization is accompanied by an increase in Cs^+^ mobility. Crystal structure model for aluminized vermiculite is based on the interstratification of unaltered vermiculite layers and aluminized layers within the same particle. Cs^+^ in vermiculite layers is poorly mobile, while the extractability of Cs^+^ is greatly enhanced in aluminized layers. The overall reactivity of the weathered clay (cation exchange capacity, Cs^+^ mobility) is then governed by the relative abundance of the two types of layers. The proposed layer model for aluminized vermiculite with two coexisting populations of caesium is of prime importance for predicting the fate of caesium in contaminated soil environments.

Existing studies devoted to the fate of radioactive caesium in polluted soil environments due to nuclear plant accidents systematically suggest that strong interactions occur between this pollutant and clay minerals, especially vermiculite-type minerals^1^,^2^,^3^,^4^,^5^,^6^,^7^,^8^,^9^,^10^. The layered structure of vermiculite involves an octahedral sheet sandwiched between two tetrahedral sheets. Isomorphic substitutions by less charged cations in tetrahedral sheets induce a permanent negative charge. This charge is compensated by the presence of exchangeable interlayer cations, which produces the high cation exchange capacity (CEC) values of vermiculites and their strong reactivity in cation adsorption/desorption processes.

In the Fukushima area, Cs^+^ is preferentially adsorbed in vermiculite-type minerals derived from the weathering of micaceous minerals^11^,^12^. However, in acidic soil environments, such as those encountered in the Fukushima area, aluminium is released into solution by the partial dissolution of vermiculite or micaceous minerals and is then commonly adsorbed again and partially hydroxylated in the interlayer space of the mineral^13^. This aluminization process in turn strongly impacts the reactive properties of the vermiculite minerals by leading to a decrease in the CEC^14^,^15^,^16^. The effects of acidic weathering and the associated aluminization of vermiculite in soils on the interactions with Cs^+^ remain poorly understood. Indeed, existing studies on this topic have either evoked an increase in Cs^+^ selectivity^1^,^9^,^17^,^18^ or a decrease in the amount of poorly mobile Cs^+ ^6,19,20,21,22. Interpretations have been systematically based on the conceptual existence of specific adsorption sites (wedge zones or frayed-edge sites, FESs) controlling the amount of Cs^+^ fixed in the mineral. Contrastingly, studies on both natural soils developed in acidic conditions^23^,^24^ or on aluminized vermiculite produced under laboratory conditions^25^,^26^ have evidenced the heterogeneous nature of the crystal structure of aluminized clay minerals. In particular, detailed analysis of experimental X-ray diffraction (XRD) patterns revealed that the crystal structure of weathered clay minerals in acidic conditions was based on the interstratification of layers with different compositions such as illite, smectite, vermiculite, and hydroxy-interlayered (HI) layers^27^,^28^,^29^,^30^,^31^,^32^,^33^,^34^. Although the interstratified nature of aluminized vermiculite has been described in literature previously, no clear quantitative relationship between the crystal structure and the mobility of Cs^+^ has been established up to now.

In this study, we propose a crystal structure model of laboratory-weathered vermiculite under acidic conditions, taking into account the actual interstratified nature of the layers. This quantitative structural description is used to provide a physical mechanistic model of Cs^+^ mobility in aluminized vermiculite. The model is based on the relative proportions of vermiculite and HI layers in the crystals and on their respective Cs^+^ extractability as determined from the combined use of XRD profile modelling and chemical measurements.

## Results

### Experimental aluminization of natural vermiculite

**Figure 1:**
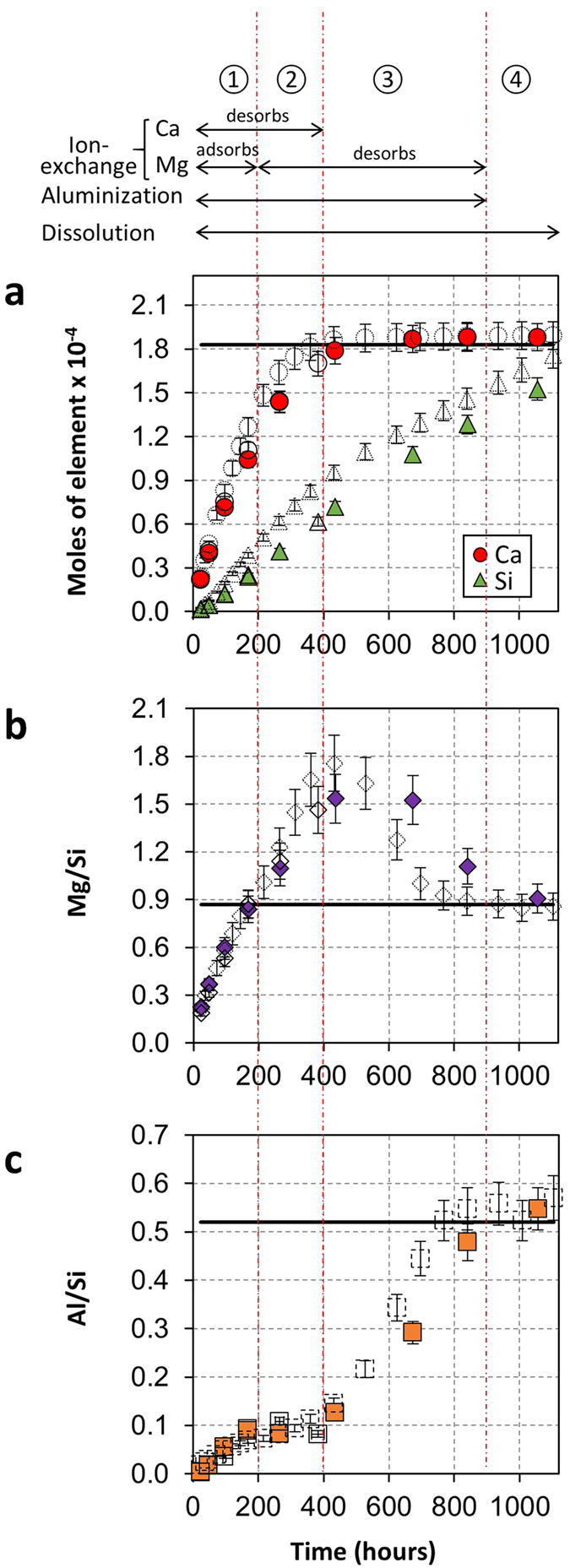
Aqueous concentrations of elements released during experimental aluminization process in acidic conditions. Aqueous Ca (circles) and Si (triangles) concentrations are shown as cumulative moles of elements (for 200 mg of vermiculite sample) in the output solutions from 0 to 1055 h (**a**). Aqueous concentrations of Mg (diamonds) and Al (squares) elements released into solution are given as normalized values relative to the dissolved Si contents, (**b,c**), respectively. The data obtained in this study (solid symbols) are compared to the analysis reported recently^35^ for the same material, setup and conditions (open symbols). In (**a**), the horizontal solid line indicates the cation exchange capacity of the material, whereas for (**b**,**c**) the horizontal lines represent dissolution stoichiometry values for Mg and Al. The top of the figure lists the different concomitant processes (dissolution, cation exchange and aluminium fixation) occurring during weathering of vermiculite.

The natural vermiculite (10–20 μm) was made homoionic via Ca^2+^-saturation in order to separately follow interlayer exchange and layer dissolution during the experimental weathering process of vermiculite under acidic conditions (HCl solution with pH = 3.0 at 25 °C). Figure 1 shows the temporal behaviour of the release of Al, Ca, Mg and Si in solution during acidic alteration of the natural vermiculite. The obtained data are close to chemical analyses reported recently for the same 10–20 μm fraction of Ca^2+^-saturated vermiculite, setup and conditions^35^, thus showing the good reproducibility of the experimental weathering process. For most silicates, the alteration process in acidic weathering involves the dissolution of the mineral, which is best revealed by the increase in cumulative aqueous Si concentrations (Fig. 1a). For swelling clay minerals, the alteration process also involves cation exchange reactions between the original interlayer Ca^2+^ ions and elements in the solution, such as protons or other elements resulting from the dissolution of the mineral itself (e.g., Al and Mg). The total amount of original interlayer Ca^2+^ is exchanged after 400 h of experiment, as shown by the plateau reached at 1.88∙10^−4^ mol (Fig. 1a) and corresponding to the initial CEC of the natural vermiculite (183 ± 13 meq/100 g)^36^. The behaviour of Mg and Al elements during the weathering process is best followed by plotting the time dependence of the amounts of Mg and Al released in solution, normalized by the dissolved Si contents (Fig. 1b,c, respectively). The obtained Mg/Si and Al/Si ratios are then compared to the dissolution stoichiometry values for both elements (DS_Mg_ and DS_Al_, respectively) calculated as the ratio between the elements from the structural formula of the solid. Calculation of saturation index of the solution towards Al-bearing phases (kaolinite, gibbsite) was performed and allowed discarding precipitation of these minerals during the acidic alteration (See Supporting Information for details of the calculation of saturation index). Then, by considering these results, X/Si < DS_X_ (with X = Al or Mg) indicates interlayer adsorption of the element X, whereas X/Si > DS_X_ indicates interlayer release of X from the vermiculite. Finally, for X/Si = DS_X_, the amount of X released in solution is stoichiometric. During the first 200 h of the experiment, both Al and Mg are adsorbed in the interlayer space of vermiculite (Mg/Si < DS_Mg_ and Al/Si < DS_Al_; Fig. 1b,c). Between 200 and 900 h of alteration, a contrasting behaviour is noticed between the two elements. As Mg/Si > DS_Mg_, the Mg adsorbed during the first 200 h in the interlayer space of vermiculite is released in solution, while Al continues to be fixed (Al/Si < DS_Al_). After 900 h of alteration time, both the Mg/Si and Al/Si ratios reach stoichiometry values (Mg/Si = DS_Mg_ and Al/Si = DS_Al_; Fig. 1b,c). This indicates that only dissolution process occurs after 900 h and that cationic exchange processes, including Al fixation, have ended. Transformation of natural vermiculite into aluminized vermiculite has thus been achieved in the experiment.

### Caesium extractability measurements in aluminized vermiculites

The two alteration experiments stopped at 383 and 1055 h correspond to two specific steps of the weathering process. For the first one, all the initial interlayer Ca^2+^ were exchanged by protons or other elements resulting from the dissolution of the mineral itself (e.g., Al and Mg), whereas the second experiment corresponds to the achievement of vermiculite aluminization. The fixed Al content in vermiculite interlayers (Table 1) is obtained from the difference between the amount of cumulative Al expected from the sole stoichiometric dissolution of the mineral and the cumulative Al measured in solution at the end of each experiment^37^. The fixed interlayer Al values are reported in Table 1 together with Cs^+^ extractability measurements, corresponding to the ability of this cation to be expelled from initially Cs^+^-saturated solid. The negligible amount of extractable Cs^+^ for the non-weathered vermiculite highlights the strong tendency of Cs^+^ to be trapped in vermiculite interlayers^38^. For altered samples, extractable Cs^+^ becomes significant and increases with fixed interlayer Al content, thus clearly showing the control of the aluminization process on Cs^+^ mobility.

### Crystal structure analysis of aluminized vermiculites

XRD analysis was used to obtain information regarding the evolution of the crystal structure as a function of the weathering process and after caesium adsorption. Due to the contrasting layer-to-layer distances between altered (i.e., HI layers) and original vermiculite layers after saturation with Na^+^, quantitative analysis of 00ℓ reflections can reveal the relative abundance of HI layers and thus the extent of the weathering process.

**Figure 2:**
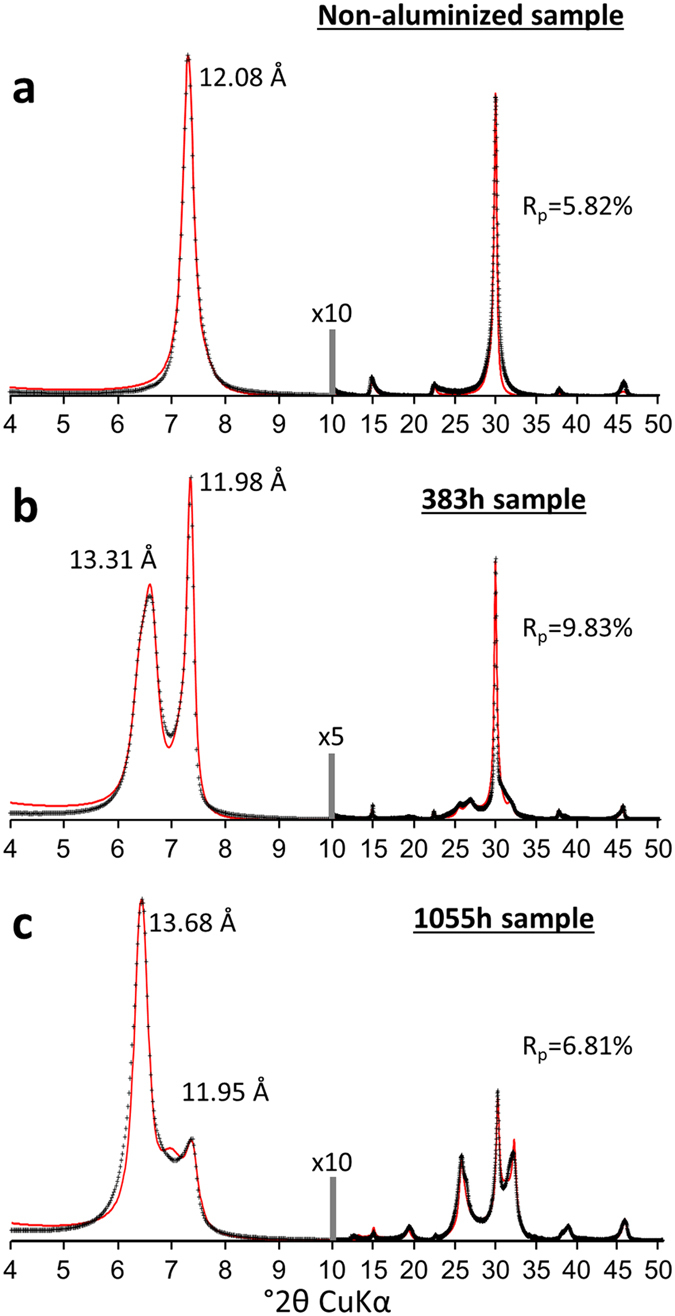
Comparison of diffraction data with the interstratified layer model of aluminized vermiculite for Na^+^-saturated specimens. Experimental diffraction patterns (crosses) are compared to calculated profiles (solid lines) for original vermiculite (**a**) and altered vermiculite in acidic conditions after 383 h (**b**) and 1055 h (**c**). The theoretical patterns account for the interstratification of non-altered and aluminized vermiculite layers in different relative proportions (See Supporting Information for details of the fitting strategy). The vertical grey bars indicate a modified intensity scale factor for the high-angle regions of the patterns compared to the low-angle regions. R_p_ parameters (the goodness of fit) are indicated for each pattern.

Figure 2 shows the experimental XRD patterns of the 00ℓ reflections for Na^+^-saturated structures of weathered vermiculites. Compared to original vermiculite (Fig. 2a) showing a 001 reflection at ~12 Å, the weathering process induces the emergence of a peak located at ~14 Å, the characteristic layer-to-layer distance for the HI layer, which increases in intensity with alteration time (Fig. 2b,c). Analysis of peak positions and shapes for the entire 00ℓ reflection series can provide additional information on the structural order/disorder in the samples. Indeed, for the case of crystals composed of layers having a unique set of layer-to-layer distances (periodic structure), a series of 00ℓ reflections is called rational, i.e., the ℓ**·***d*_00ℓ_ product is a constant for all reflections. In the case of crystals composed of a random interstratification of layers with contrasting layer-to-layer-distances (e.g., 12 and 14 Å), the resulting diffraction pattern will display peaks in intermediate positions between those expected for periodic 12 or 14 Å structures. Moreover, the peak widths of the resulting signal will depend not only on the mean coherent scattering domain size but also on the angular distance between the positions expected for reflections of periodic end-members^39^. This diffraction effect on peak position and shape is known as Méring’s rule^40^ and is responsible for the loss of rationality in the series of 00ℓ reflections (i.e., variation in the ℓ**·***d*_00ℓ_ product from one reflection to another). For the experimental XRD patterns of weathered materials (Fig. 2b,c), the reflection near 12 Å remains during the alteration process. However, during alteration, the peak near 14 Å increases in intensity and shifts in position towards higher *d*_001_ values. This evolution is accompanied by significant variations in peak positions and widths for other 00ℓ reflections. Both effects suggest the presence of interstratified structures for weathered vermiculites. The presence of an interstratified structure implies the need for the use of specific XRD calculation routines, such as those based on a physical description of the distribution of layers in crystals using Markovian statistics^39^,^41^.

**Figure 3:**
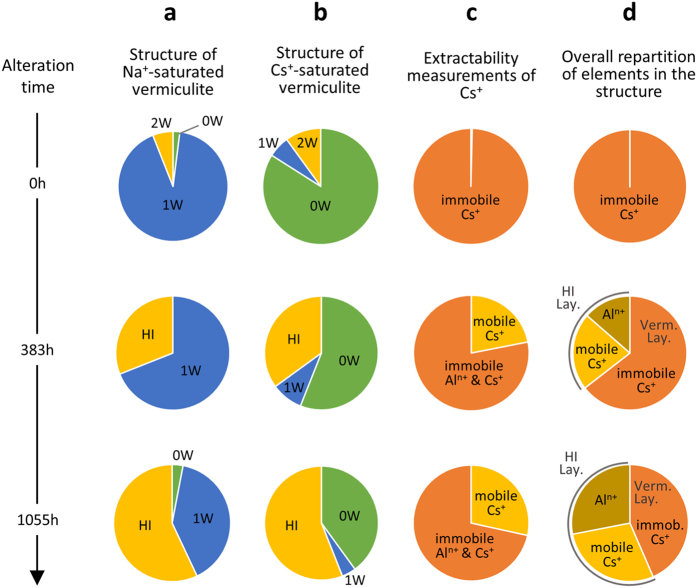
Quantitative structure model for Cs^+^ distribution in aluminized vermiculite. (**a,b**) correspond to the relative proportions of dehydrated (0 W), monohydrated (1 W), bi-hydrated (2 W) or hydroxy-interlayered (HI) layers obtained from XRD analysis after Na^+^ or Cs^+^ saturation of the samples, respectively, as a function of alteration time under acidic conditions (0 h, 383 h and 1055 h from top to bottom in the figure). (**c**) Relative proportions of mobile and immobile Cs^+^ obtained from extractability measurements (Table 1). (**d**) Quantitative distribution model of Al(OH)_x_^3−x^ islands, mobile Cs^+^ in hydroxy-interlayered layers and immobile Cs^+^ located in vermiculite layers as a function of the weathering process of vermiculite under acidic conditions.

**Figure 4:**
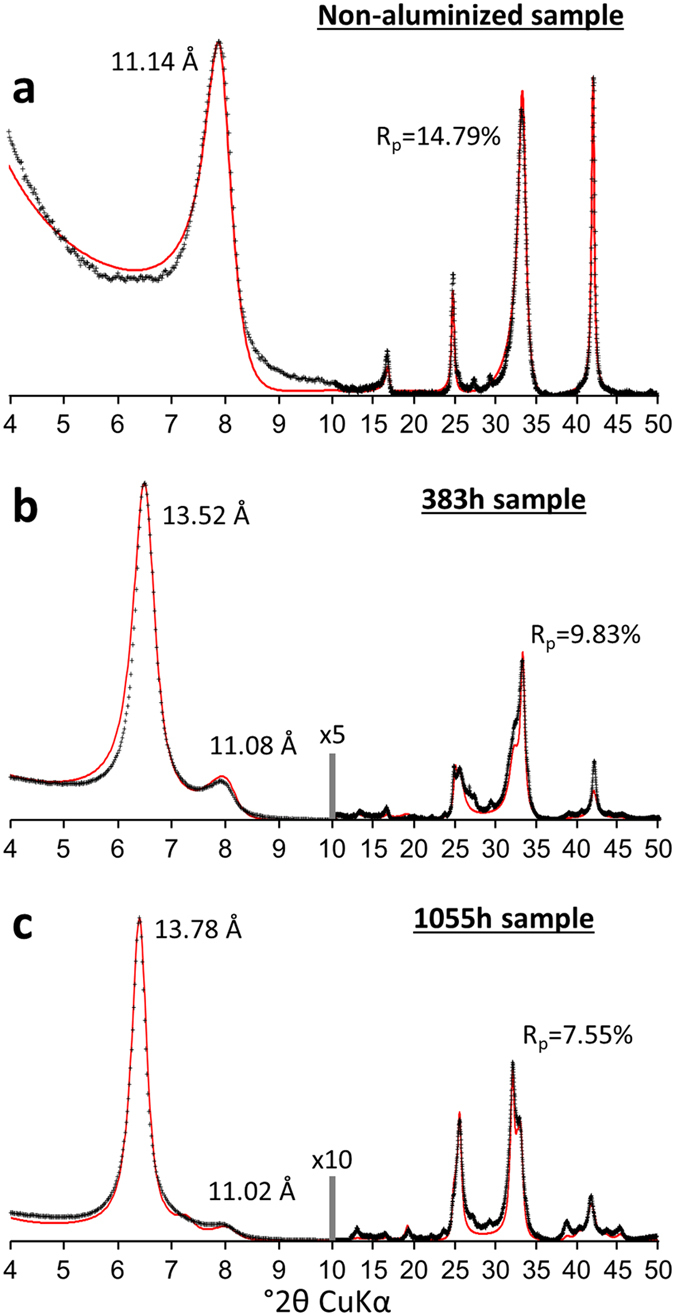
Comparison of diffraction data with the interstratified layer model of aluminized vermiculite for Cs^+^-saturated specimens. The fits are performed for XRD patterns collected after Cs^+^ saturation of the samples shown in Fig. 2. Same notations and symbols as in Fig. 2 for original vermiculite (**a**) and vermiculite altered under acidic conditions for 383 h (**b**) and 1055 h (**c**).

Figure 2 also includes a comparison between experimental patterns and calculated XRD profiles accounting for the interstratified nature of the obtained structures. These calculations are based on the consideration of three types of layers characterized by contrasting layer-to-layer distances. Two of them correspond to two classical hydrates of vermiculite with zero or one water layer (0 W: *d*_001_ ~ 10 Å and 1 W: *d*_001_ ~ 12 Å, respectively) whereas the third type of layer corresponds either to a bi-hydrated state of vermiculite or to HI layers (2 W/HI: *d*_001_ ~ 14 Å). Details on the composition of the different interstratified structures and their proportions used for the modelling procedure are reported in the Supporting Information. Based on the fitting procedure, the relative proportions of different layers characterized by contrasting layer-to-layer distances for non-weathered and weathered vermiculites are reported in Fig. 3a. The original Na^+^-saturated vermiculite structure displays an overwhelming contribution of 1 W layers with small amounts of 2 W and 0 W layers as a result of hydration heterogeneities, as shown repeatedly in the literature^25^,^42^. For simplicity, all layers with a *d*_001_ of ~14 Å in altered samples are attributed to HI layers. With alteration time, the increase in the relative abundance of HI layers is consistent with the increase in intensity of the peak located at ~14 Å (Figs 2 and 3a) and consequently with the ongoing aluminization process of vermiculite. After Cs^+^ saturation (Fig. 4), the experimental XRD patterns for the same samples show that the reflection near 14 Å remains for both altered samples whereas the peak located at ~12 Å for Na^+^-saturated specimens shifts towards ~11 Å. Quantitative results from the fit of XRD patterns (Fig. 4; See Supporting Information for details of the fitting procedure) indicate that this ~12-to-11 Å peak shift is related to the transformation of 1 W into 0 W layers related to the Cs^+^-for-Na^+^ exchange. The relative abundance of HI layers remains nearly constant, regardless of the cation exchange process applied to the altered samples (Fig. 3a,b).

**Figure 5:**
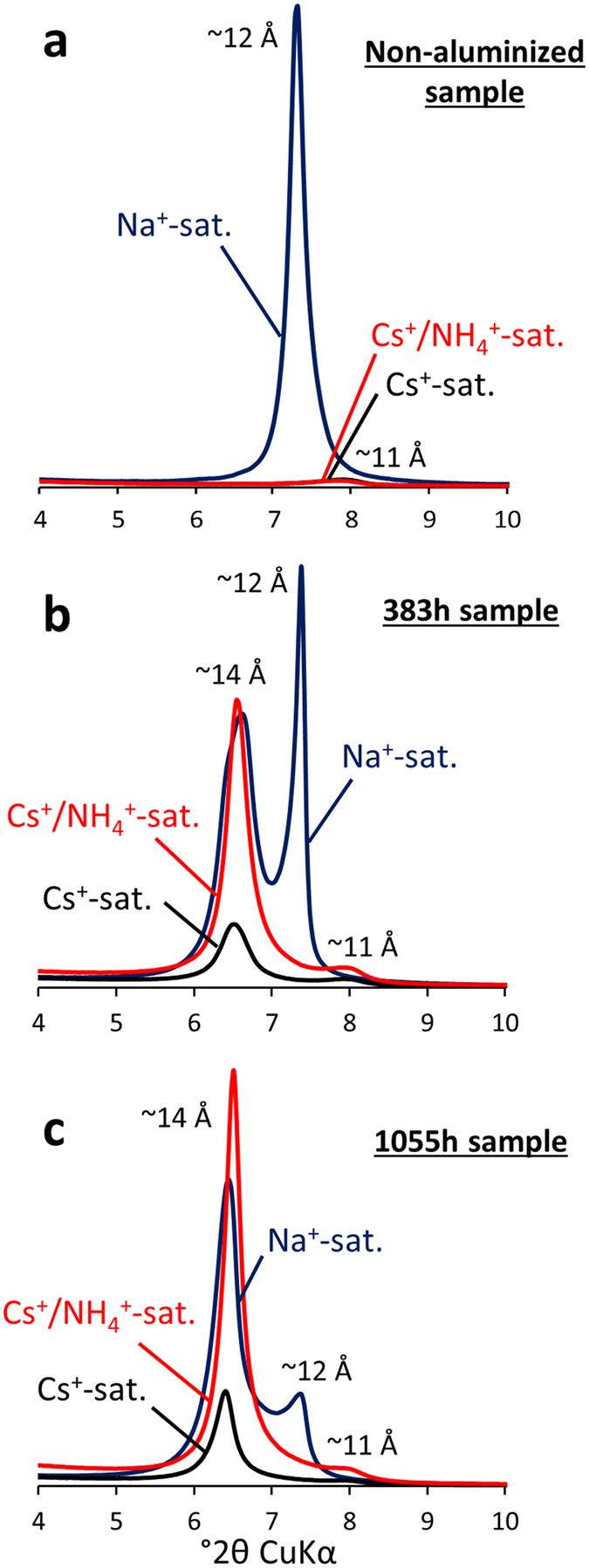
Evidence of Cs^+^ adsorption in aluminized vermiculite layers. Qualitative comparison of experimental diffraction patterns obtained after different sample treatments for original vermiculite (**a**) and vermiculite altered under acidic conditions for 383 h (**b**) and 1055 h (**c**). Blue: Na^+^-saturated specimen (Na^+^-sat.). Black: Cs^+^-saturated sample (Cs^+^-sat.). Red: after NH_4_^+^-exchange of Cs^+^-saturated specimen (Cs^+^/NH_4_^+^-sat.). The limited modification in the intensity of the peak at ~11 Å between Cs^+^ saturation and Cs^+^/NH_4_^+^ saturation of the samples indicates the presence of immobile Cs^+^ in the vermiculite layers. The change in intensity of the peak at ~14 Å after the different treatments provides evidence of the presence of a remaining layer charge in hydroxy-interlayered layers with reversible adsorption sites for Cs^+^.

Information regarding the presence of Cs^+^ cations in the different types of layers can be obtained through a qualitative comparison between experimental XRD patterns collected after Na^+^ and Cs^+^-exchange. These two patterns are also compared to the profile obtained after NH_4_^+^ saturation of Cs^+^-exchanged samples (i.e., Cs^+^/NH_4_^+^ -samples; Fig. 5). To allow qualitative comparison of the 001 reflection intensities for the different treatments, particular care was taken to prepare and analyse the samples in the same way (i.e., same sample mass, length, sample preparation method and XRD acquisition parameters). In addition to a change in the layer-to-layer distance (i.e., 1 W to 0 W transition) in the non-altered original vermiculite (Fig. 5a), Cs^+^ saturation of Na^+^-vermiculite induces a significant peak intensity change in the low-angle region of the XRD patterns. As recently discussed^43^, this decrease in intensity of the 001 reflection for Cs^+^-saturated samples is related to the change in the layer structure factor F(θ), which can be calculated for the 00ℓ reflections as follows:


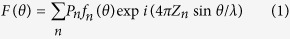


where θ is the scattering angle and λ is the wavelength of the X-ray radiation. Parameters P_*n*_ and Z_*n*_ stand for the amount and position along c* axis, respectively, of the atom *n* in the unit-cell. Finally, parameter *f*_*n*_(θ) corresponds to the atomic scattering power of the atom *n*, which is based on its number of electrons. The intensity of the 00ℓ reflections will depend on the product of this function and its conjugate: *I* = *F*(θ)∙F*(θ)^39^,^41^. Accordingly, for a sample constituted by layers having the same layer charge and layer-to-layer distance, the intensity of the 001 reflection will mainly be affected by the nature of the atoms in the unit-cell and their respective number of electrons (related to the *f*_*n*_(θ) parameter). When cation exchange is the sole process impacting the structure and the layer-to-layer distance remains unchanged, the change in the intensity of the 001 reflection can be attributed to the value of the parameter *f*_*n*_(θ) of the interlayer cation. As seen in Fig. 5a, the intensity of 001 reflections for Cs^+^-saturated vermiculite (Cs^+^-sat.) before and after treatment with NH_4_^+^ (Cs^+^/NH_4_^+^-sat.) are exactly the same. This indicates that NH_4_^+^ ions did not penetrate the interlayer space of vermiculite. Indeed, Cs^+^ and NH_4_^+^ hold 54e^−^ and 10e^−^, respectively, which in turn induce very different *f*_*n*_(θ) values in equation (1). Accordingly, in the case of a complete NH_4_^+^-for-Cs^+^ exchange, one would expect an increase in the intensity of the 001 reflection by a factor of 20 between Cs^+^- and NH_4_^+^-saturated vermiculites^43^. For altered vermiculite samples (Fig. 5b,c), the shift of the ~12 Å peak towards higher angles (i.e., down to ~11 Å) and its decrease in intensity between XRD patterns collected after Na^+^ and Cs^+^ treatment are again related to the change in the layer structure factor (equation 1). The obtained peak at 11 Å under Cs^+^-treatment is not modified in position and shape after NH_4_^+^ -exchange (i.e., Cs^+^/NH_4_^+^ samples) but slightly shifts in intensity due to the concomitant increase of the 14 Å reflection high-angle tail. This is consistent with the presence of non-exchangeable Cs^+^-saturated layers with the same properties as original vermiculite. Contrastingly, the ~14 Å peak displays an important change in intensity after the different treatments (i.e., Na^+^, Cs^+^ and NH_4_^+^ exchange). Indeed, although the position of this peak remains unaffected by the cation exchange processes, the intensity dramatically decreases between Na^+^- and Cs^+^-saturated specimens (Fig. 5b,c). This indicates that adsorption of Cs^+^ has occurred in the interlayer space of the HI layers. More interestingly, the intensity of this contribution at ~14 Å increases again after Cs^+^/NH_4_^+^ exchange, suggesting the removal of the Cs^+^ originally located in these HI layers. These findings indicate the coexistence of two populations of Cs^+^, associated with two different types of layers (HI and original vermiculite layers, respectively) and having contrasting mobility. Poorly exchangeable Cs^+^ can be attributed to the original vermiculite layers, which remain unaffected by the different treatments applied (i.e., Na^+^, Cs^+^ and NH_4_^+^ exchange), whereas highly exchangeable Cs^+^ is attributed to the HI layers, which increase in proportion with alteration time.

### Quantitative structure model for caesium distribution in aluminized vermiculite

**Figure 6:**
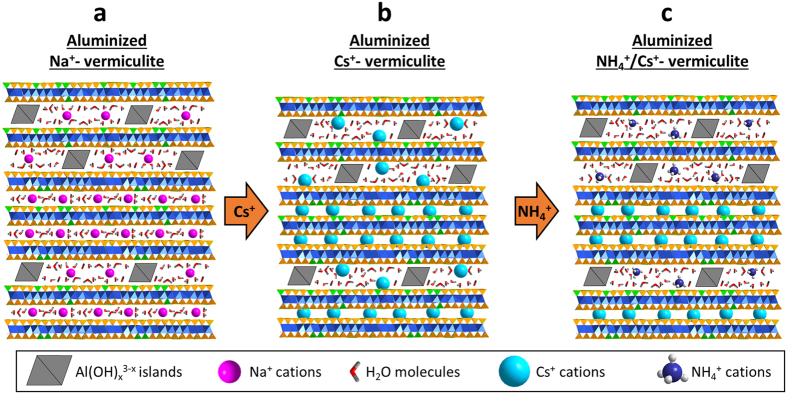
The layer model for aluminized vermiculite structure and implications for Cs^+^ mobility. (**a**) Aluminized vermiculite is composed of an interstratification of unaltered Na^+^-saturated vermiculite layers and hydroxy-interlayered layers containing both Al(OH)_*x*_^3−*x*^ islands and Na^+^ cations. (**b,c**) For unaltered vermiculite layers, Cs^+^ cations are immobilized by the collapse of the structure whereas Cs^+^ remains mobile in the hydroxy-interlayered layers and exchangeable with NH_4_^+^ ions.

The combination of structural and chemical data can be used to develop a comprehensive structural model of aluminized vermiculite interpreting Cs^+^ mobility. This general structural model is schematized in Fig. 6. The XRD analyses show the disordered nature of the aluminized structure with significant interstratification of HI layers and non-altered layers possessing the same properties as the original vermiculite sample (Fig. 6a). Whereas HI layers can exchange Cs^+^, non-altered vermiculite layers collapse after Cs^+^-exchange (Fig. 6b). This collapse in turn leads to interlayer Cs^+^ that cannot be easily desorbed by NH_4_^+^ (Fig. 6c).

In addition, XRD analysis revealed a constant proportion of HI layers, regardless of Na^+^ or Cs^+^ saturation but increasing in abundance with alteration time (Fig. 3a,b). This increase in HI layers content with alteration time is correlated with the overall rise in the amount of extractable Cs^+^ (Fig. 3c, Table 1). Finally, an assessment of the composition of HI layers can be performed by compiling both structural and chemical data. The total layer charge^44^, considered a constant through the alteration process, is distributed between non-altered and HI layers, based on the mean relative proportions deduced using XRD profile modelling after Na^+^ and Cs^+^ saturation (Fig. 3a,b). The amount of charge associated with specific HI layers is then distributed between caesium and aluminium (Table 1). In agreement with structural data (Figs 5 and 6c), experimental extractable Cs^+^ (Fig. 3d, Table 1) is assigned to HI layers. Considering the remaining layer charge and the estimate of Al interlayer content (Table 1) in HI layers, the calculation leads to a lower charge compared to the +3 charge expected for aluminium (i.e., a charge at ~1.4 and 2.3 per Al for the samples collected at 383 and 1055 h, respectively). This provides additional support for the interstratified structure model of aluminized vermiculite proposed here, in agreement with the presence of Al(OH)_x_^3−x^ islands, as proposed previously^25^,^29^,^45^.

## Discussion

The experimental aluminization of natural vermiculite performed in this work accurately represents the mechanisms encountered in a natural weathering context due to presence of dissolution, cation exchange and aluminium fixation (Fig. 1). The resulting weathered vermiculite contains both original vermiculite layers and newly formed HI layers. These two types of layers are interstratified in the same crystals leading to strongly heterogeneous structures (Fig. 6). Although this complex crystal structure has been evoked in the past in both experimental alteration experiments and for natural soil samples^27^,^28^,^30^,^31^,^32^,^33^,^34^,^46^, the structural quantification of these two types of layers has been performed only very recently^25^,^26^. Moreover, such a quantitative structural model based on interstratification of layers has not yet been used to explain the mobility of Cs^+^ associated with clay minerals.

We propose here a new type of crystal structure model of aluminized vermiculite including both the actual interstratified nature of layers and the presence of different populations of Cs^+^ with contrasting mobility (Fig. 6). Vermiculite layers unaltered by acidic conditions are associated with poorly exchangeable Cs^+^ by NH_4_^+^. For HI layers, the amount of adsorbed Cs^+^ decreases due to the presence of fixed Al(OH)_x_^3−x^ islands; however, once adsorbed, the Cs^+^ remains extractable. According to this model, the overall properties of the material are governed by the relative abundance of these two types of layers. Note that in line with the large size fraction used in the present study, the amount of sorption sites on the external surfaces of the particles can be considered as negligible^47^. With progression of the aluminization process, the increase in the proportion of HI layers at the expense of the original vermiculite layers induces a decrease of the amount of exchangeable cations but an overall increase in the amount of extractable Cs^+^ (Table 1).

This layer model is also consistent with the experimental studies based on weathered biotite from Fukushima^48^. For CsCl solutions of 1.5∙10^−3^ mol/L concentrations, these authors indeed demonstrated using high-resolution transmission electron microscopy that Cs^+^ infiltrated deeply into the vermiculite interlayers. For Cs^+^ adsorption at trace concentrations, Maes *et al*.^20^ showed that fixation of Cs^+^ increases after the removal of interlayer Al from natural soil samples containing aluminized vermiculite. In line with this observation, Maes *et al*.^21^ also reported that the relative proportion of fixed Cs^+^ at trace concentrations of 1∙10^−10^ mol/L is drastically lower after the experimental aluminization of vermiculite relative to the original vermiculite sample. Moreover, the subsequent removal of interlayer Al from aluminized vermiculite leads to a recovery of the original Cs^+^ fixation properties of the sample. Although the mechanism of caesium fixation may differ depending on the concentration of Cs^+^ chosen, i.e., interlayer collapse in the present study vs. specific site caesium fixation in the case of trace concentrations^21^, both studies show qualitative agreement on the influence of aluminization on the Cs^+^ extractability.

In conclusion, existing studies on polluted soil samples from Fukushima have revealed the intimate relationship between the fate of Cs^+^ and the clay minerals present in the soil, especially vermiculite-type minerals^10^,^12^,^49^,^50^. However, acidic conditions, such as those encountered in soils in the Fukushima area, lead to the weathering of the original clay material and the formation of aluminized clay minerals^8^,^19^. Here, we propose a comprehensive structural model for aluminized vermiculite based on the interstratification of both vermiculite and HI layers with differing Cs^+^ extractability properties. This layer model should help in refining the role played by weathered clay minerals in the fate of Cs^+^ in contaminated soils. Accordingly, this study also stresses the need for more in-depth crystal structure analysis of natural clay minerals from the Fukushima area, taking into account their heterogeneous nature in terms of layer composition, abundance and interstratification.

## Material and Methods

### Materials

Natural vermiculite from Santa Olalla with the chemical composition (Mg_0.75_Ca_0.05_Na_0.04_)(Mg_4.92_Al_0.59_Fe_0.43_Ti_0.04_)(Si_5.66_Al_2.34_)O_20_(OH)_4_ 44 was used. First, 1–4 mm sized crystals were obtained by dry sieving, then purified by removing magnetic components and by selecting non-altered crystals on a light table. Then, the crystals were immersed in 10^−4 ^mol/L HCl for 5 min to remove carbonate minerals. The pre-treatment procedure was concluded by rinsing the crystals 3 times with distilled water to remove the acid solution. To break down the crystals, an ultrasound probe was used. Then, 3 g of 1–4 mm purified vermiculite crystals were sonicated in 50 mL of distilled water for 10 h in continuous mode. Size fractioning of sonicated material was performed by sieving, centrifugation and sedimentation. To produce 10 g of material in the 10–20 μm size fraction with this protocol required an initial mass of 123 g of 1–4 mm crystals. After fractionation, 10–20 μm vermiculite particles were saturated with Ca^2+^ to obtain a homo-ionic sample. The material was put in contact with the 1 mol/L CaCl_2_ solution for ~12 h (m/V = 5 g/L). This procedure was repeated five times using successive solid and solution separation steps via centrifugation and the addition of fresh saline solutions. After saturation, the material was rinsed with ultrapure water (18 MΩ. cm) until a silver nitrate test for Cl^−^ was negative and then air-dried at room temperature.

### Aluminization experiments

The experiment was performed at 25 °C using 18 mL flow-through reactors. For each reactor, two replicates containing 200 mg of vermiculite were performed. An acid solution of HCl (pH = 3.0) at a flow rate of 0.046 mL/min was continuously injected through the reactor by using a peristaltic pump. Periodic sampling at 23, 48, 96, 168, 266, 436, 673, 841, 1055 hours was performed to document the rate of element release in the solution and to calculate the Al in the interlayer at the end of alteration experiment.

The Al, Fe, Ca, Mg and Si concentrations of the output solutions were measured with an inductively coupled plasma optical emission spectrometer (ICP-OES, Perkin Elmer Optima 2000DV). The detection limits were less than 5 ppb for all the elements, and the maximum uncertainties were ±3% for all elements based on reproducibility tests.

The solid sample of the first replicate was recovered after 383 hours (16 days), and the solid sample of the second replicate was recovered after 1055 hours (44 days). The recovered solid samples were filtered on a 0.1 μm cellulose nitrate filter, rinsed several times with ultrapure water and then air-dried.

The dissolution of the material was calculated based on dissolved cumulative Si amount. Interlayer Al was calculated by subtracting the measured cumulative amount of released Al in solution from the total amount leached from the structure in relation to the Si released assuming stoichiometric dissolution. The contribution of Fe was not taken into account in the calculations as it represented a minor contribution.

### Cs^+^ extractability measurements

After the alteration, 75 mg of the sample were saturated with a 1 mol/L NaCl solution to exchange all non-fixed interlayer species, and then 25 mg of Na^+^-saturated samples were exchanged with a 1 mol/L CsCl solution. In both cases, the solid-solution ratio was 2.5 g/L. The saturation process was performed 5 times, renewing the salt solution after 12 hours of contact time. The resulting Na^+^- and Cs^+^-saturated altered vermiculite samples were washed 3 times with ultra-pure water (18 MΩ·cm) and then air-dried. To assess the amount of extractable Cs^+^, Cs^+^-saturated samples were exchanged with 1 mol/L ammonium acetate solution. For this, 25 mg of sample were put in contact with 1 mol/L ammonium acetate solution for 3 days on a mechanical shaker. Afterwards, the samples were centrifuged and aqueous Cs^+^ concentrations were measured in an aliquot of the supernatant with atomic absorption spectroscopy (AAS, Varian AA240FS). The aliquot of the sample was diluted in 2% HNO_3_ in order to measure aqueous concentrations between 0.5 and 5 mg/L, corresponding to the linear ranges of the calibration curves. To account for possible interferences during measurements, samples and standards were prepared in 2 g/L KCl solutions. The total uncertainties in the measured concentrations of all of the cations were estimated to be ±2%.

### XRD experiments

The altered mineral structure was analysed using X-ray diffraction (XRD). To distinguish altered (HI) and non-altered layers, the samples were first Na^+^-saturated and XRD pattern acquisition was performed at a fixed low value of relative humidity (RH). Indeed, it has been shown previously^25^ that, by working at low RHs (<40%), it is possible to maximize the layer-to-layer distance contrast between Al and Na^+^-saturated interlayers. Thus, the data collection was performed at 30% RH, after an equilibrium period of 20 min.

XRD experiments were performed on oriented preparations in order to assess the extent of interlayer aluminization. To obtain these oriented preparations, an aliquot of the clay dispersion was dropped onto a glass slide and dried at room temperature.

The XRD patterns were recorded using a Panalytical X’Pert Pro MPD diffractometer equipped with an X’Celerator detector operating with an angular aperture of 1.021° and a VTI RH-100 humidity generator device coupled to an Anton Paar THC chamber. The scanning parameters were 0.033°2θ for the step size and 6 s for the counting time per step throughout the 2–50°2θ CuK_α1+2_ angular range. The divergence slit, the anti-scatter slit and the two Soller slits were 0.125°, 0.25°, 2.3° and 2.3°, respectively.

### XRD profile modelling of 00ℓ reflections

The algorithms developed initially by Sakharov and co-workers were used to fit experimental XRD patterns for the 4–50°2θ CuKα range using a trial-and-error approach^51^,^52^,^53^. For each XRD pattern, structural parameters, such as the composition of the interstratified structures (proportions of the different layer types), their stacking mode (Reichweite parameter R, junction probabilities), and an estimate of their relative proportions, are adjusted to fit the experimental XRD pattern. Instrumental and experimental factors, such as horizontal and vertical beam divergences, goniometer radius, and length and thickness of the oriented slides, were measured and introduced without further adjustment. The mass absorption coefficient (μ*) was set to 45 cm^2^/g, as recommended by Moore and Reynolds^54^. Additional variable parameters include the layer-to-layer distance of hydrated layers and the coherent scattering domain size (CSDS) along the c* axis, characterized by a lognormal distribution around a variable mean value (N) and a maximum CSDS value set to 80 layers^55^.

The layer-to-layer distance was allowed to deviate from its mean value by introducing a variance parameter σ_z_ (from 0.10 to 0.18 Å) to account for this “disorder of the second type”^39^,^56^. The z-coordinates for all atoms within the 2:1 layer framework were set to those proposed by Moore and Reynolds^54^. Chemical composition of the 2:1 layer framework was considered as constant through the alteration experiment^25^.

The interlayer configuration used for bi-hydrated (2 W) and hydroxy-interlayered (HI) layers were those proposed initially by Ferrage *et al*.^56^ and Lanson *et al*.^25^, respectively, with one plane of H_2_O molecules on each side of the interlayer mid-plane that hosts cations. This model is characterized by a distance (Δd2W) between the interlayer mid-plane and each of the planes of H_2_O molecules, which was set to 1.20 Å. The layer-to-layer distance for 2 W of non-altered Na^+^ -vermiculite was set to ~14.8 Å, consistent with the value of ~14.7 Å reported by de la Calle *et al*.^57^ For altered samples, the HI layer-to-layer distance was set to ~14.0 Å, corresponding to the previously reported value of Lanson *et al*.^25^. For monohydrated layers (1 W, *d*_001_ ~ 12.0 Å), both cations and H_2_O molecules were located in the interlayer mid-plane. A second 1 W layer type was used in the case of Na^+^-saturated samples of ~12.5 Å^58^. A similar configuration was used for dehydrated layers (0 W) without interlayer water molecules. For Na^+^-saturated samples, *d*_001_ = 10.0 Å, and for Cs^+^-saturated samples, *d*_001_ = 10.8 Å. The water content was allowed to vary between 3 and 5 H_2_O molecules per formula unit for 1 W layers and between 6 and 9 molecules for 2 W/HI layers. The Debye-Waller factor of H_2_O molecules was allowed to vary between 15 and 20 Å^2^ for 1 W and was set to 15 Å^2^ for 2 W layers in an effort to optimize the electronic density profile of interlayer species, as proposed by Dazas *et al*.^59^,^60^.

## Additional Information

**How to cite this article**: Dzene, L. *et al*. Crystal structure control of aluminized clay minerals on the mobility of caesium in contaminated soil environments. *Sci. Rep.* **7**, 43187; doi: 10.1038/srep43187 (2017).

**Publisher's note:** Springer Nature remains neutral with regard to jurisdictional claims in published maps and institutional affiliations.

## Supplementary Material

Supporting Information

## Figures and Tables

**Table 1:** Determination of interlayer Al contents and measured amounts of extractable Cs^+^ as a function of the weathering process.

Sample (h)	Dissolved sample (wt%)	Interlayer Al (× 10^−4^ mol/g)	Extractable Cs^+^ (× 10^−4^ mol/g)
0	0	0	0.06 ± 0.002
383	5.0 ± 0.1	1.42 ± 0.05	3.7 ± 0.1
1055	13.0 ± 1.0	2.22 ± 0.17	4.8 ± 0.2
