# Supplementary material for: Crystal structure control of aluminized clay minerals on the mobility of caesium in contaminated soil environments
Source: Sci Rep. 2017 Feb 24;7:43187. doi: 10.1038/srep43187 (PMC5324138; doi:10.1038/srep43187)
Supplement: Supporting Information [file srep43187-s1.pdf]

SUPPORTING INFORMATION FOR

Crystal structure control of aluminized clay minerals on the mobility of caesium in contaminated soil environments

Liva Dzene[a*], Eric Ferrage[a], Jean-Christophe Viennet[a,b], Emmanuel Tertre[a], Fabien Hubert[a]

[a] Université de Poitiers/CNRS, UMR 7285 IC2MP, Equipe HydrASA, 5 rue Albert Turpain, Bât. B8, TSA - 51106, 86073 Poitiers cedex 9, France

[b]Now at The Centre for Earth Evolution and Dynamics, University of Oslo, Sem Saelandsvei 24, NO-0371 Oslo, Norway

*liva.dzene@univ-poitiers.fr

Assessment of potential precipitation of Al-bearing phases during acidic weathering of vermiculite

To assess potential precipitation of Al-bearing phases during acidic alteration of vermiculite, calculation of saturation index was performed with Phreeqc® software associated with the thermodynamic database proposed by Parkhurst and Appelo[1]. The chemical composition of the solutions measured as a function of time at the outlet of the reactor were used as input parameters for these calculations. The obtained results reported in Table S1 show that solutions are systematically under-saturated with respect to Al-bearing phases, such as kaolinite and gibbsite through the entire alteration timespan.

Table S1. Chemical composition of aqueous Al, Fe, Mg, Si and Ca species as a function of alteration time and calculated saturation index (SI) values respective to kaolinite and gibbsite. SI is defined as the ionic activity product divided by the solubility product.

Time, hours	pH	Al mg/L	Fe mg/L	Mg mg/L	Si mg/L	Ca mg/L	log(SI) kaolinite	log(SI) gibbsite
23	3.59	0.003	0.017	0.142	0.72	13.760	-9.97	-4.46
48	3.39	0.021	0.046	0.389	1.22	10.510	-9.01	-4.20
96	3.37	0.083	0.098	0.805	1.55	9.481	-7.72	-3.67
168	3.31	0.140	0.134	1.190	1.64	6.517	-7.57	-3.61
266	3.35	0.145	0.184	1.720	1.81	5.852	-7.22	-3.48
436	3.28	0.223	0.262	2.430	1.83	2.993	-7.25	-3.50
673	3.19	0.429	0.269	2.020	1.53	0.481	-7.36	-3.48
841	3.13	0.570	0.159	1.190	1.24	0.110	-7.66	-3.54
1055	3.13	0.605	0.107	0.904	1.15	0.008	-7.67	-3.51

Description of the fitting strategy for the modeling of experimental XRD patterns

The experimental and calculated X-ray diffraction (XRD) patterns for the Na^+- and Cs^+-saturated non-altered and altered vermiculite are shown in Fig. S1 and Fig. S2, respectively. These experimental patterns were recorded at 30% relative humidity (RH) because Na^+- and Cs^+-saturated vermiculite display structures close to the mono-hydrated (1W) and dehydrated (0W) states, respectively, in such condition. Due to hydration heterogeneities, the samples most often includes both type of layers, however, plus additional bi-hydrated (2W) layers[2,3]. For altered vermiculite samples, the acidic conditions lead to the transformation of vermiculite layers into hydroxy-interlayered (HI) layers with a layer-to-layer distance of ~14.0 Å. For the sake of simplicity presence of 2W layers in addition to HI layers was not considered for altered samples (see materials and method section for the description of models used for the different layer types).

The fitting procedure used to reproduce experimental XRD patterns is detailed elsewhere[2,4,5]. Briefly, one periodic sequence of layers (0W, 1W, or 2W/HI only) was used first to reproduce as much as possible of the experimental XRD pattern. If necessary, extra contributions containing two or three layer types were added to the calculated profile introduced to account for the misfit between calculated and experimental patterns. Because of significant structural heterogeneity for altered samples leading to diffraction maxima with complex and asymmetric profiles, up to four mixed-layer structures (MLSs; each with different proportions of layer types) were necessary to reproduce some of the experimental patterns. Layers with the same hydration state were assumed however to have identical parameters (identical values N and σ_z parameters, and atomic content) in the different MLSs. Note that the consideration of several MLSs to fit all features of experimental XRD patterns does not imply the actual presence of different populations of particles in the sample but rather indicates that layer nature and respective hydration heterogeneities are not randomly distributed within vermiculite crystallites. Relative proportions of the different mixed-layer structures and that of the different layer types in the mixed-layer structures were considered as variable parameters in the fitting procedure. The Rp parameter[6], which is mainly influenced by fit quality of intense diffraction maxima, was used to characterize the overall fit quality.

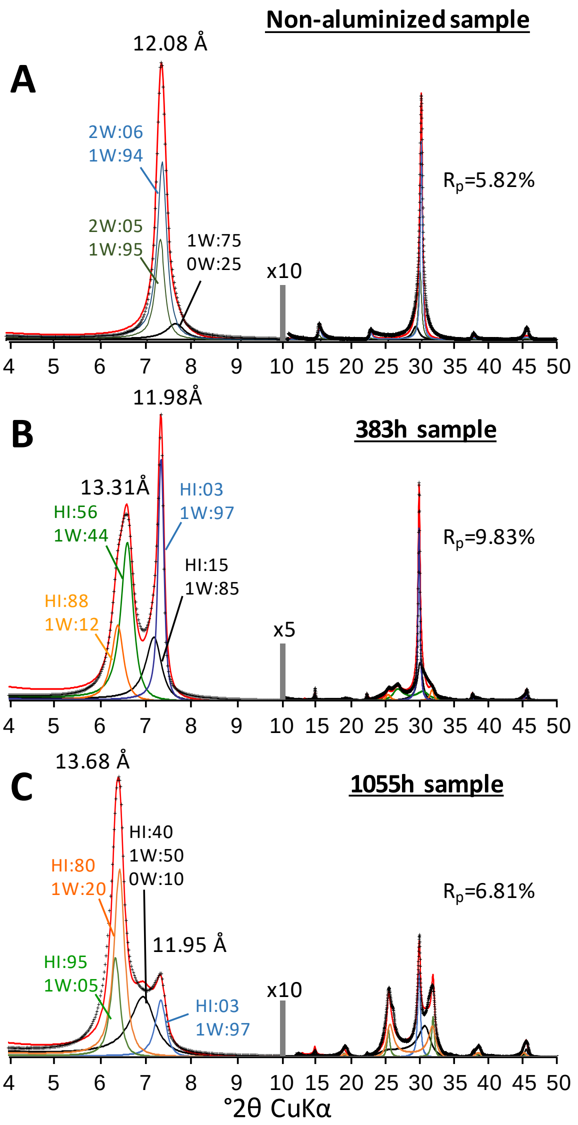

Figure S1. Comparison between the experimental (crosses) and calculated (solid red lines) XRD patterns recorded at 30% relative humidity for Na^+-saturated material. (A) Reference non-altered Na^+-vermiculite sample. (B) Sample altered for 383 hours. (C) Sample altered for 1055 hours. The vertical gray bars indicate a modified scale factor for the high-angle regions compared with the 2-10° 2θ angular range. The respective contributions of the various mixed-layer structures to the calculated profile and the goodness of fit R_p parameters are indicated for each pattern.

Calculated patterns are compared to experimental XRD profiles in Figures S1 and S2 for Na^+- and Cs^+-saturated vermiculites, respectively, as a function of alteration time. The number and composition as well as the Reichweite parameter of MLSs used in the fitting procedure are reported in Table S2. This table also includes the number N of layers in coherent scattering domain size, the layer-to-layer distances of 0W, 1W, 2W and HI layer types, and the overall relative contributions of the different layer types in the samples.

The experimental XRD pattern of the original Na^+-vermiculite (Fig. S1A) was reproduced considering three randomly interstratified (R0) MLSs. The first two MLS are mostly composed of 1W layers and minor amounts of 2W (1W:2W=94:06 and 95:05, respectively; Table S2). These two MLSs however differ due to the need of consideration of two different layer-to-layer distances for 1W layers, at ~11.9 Å and ~12.5 Å (1Wa and 1Wb, respectively; Table S2). In agreement with previous studies on this vermiculite sample[7], the consideration of these two 1W layer types, in different proportion in the two MLSs, allows reproducing most of the experimental pattern (Fig. S1A). A third MLS is necessary, however, to account for the asymmetry on the high-angle side of the 001 reflection. By combining the three MLSs in 58:30:12 proportion a satisfactorily fit of experimental pattern is obtained. Such combination leads to an overall HI/2W/1W/0W layer content of 0/5/92/3, thus confirming the overwhelming contribution of 1W layers at this RH value. The layer-to-layer distance for 2W layers of non-altered Na^+-vermiculite used in the calculations at 14.8 Å is consistent with the value reported at 14.7 Å by de la Calle *et al.*[8] for the same material. Two different layer-to-layer distances for Na^+-saturated 1W layers were used during the modeling exercise, at 11.9 Å and 12.5 Å (1Wa and 1Wb, respectively; Table SI 1), in agreement with previous studies on this vermiculite sample[7]. Finally, this sample display high N values, consistent with the high crystallinity of the sample. For the sample altered in acidic conditions during 383h (Fig. S1B), the peak related to original vermiculite at ~12 Å is still present and is accounted for in the calculations by considering two R0 MLSs dominated by 1W layers (Table S2). Both contributions also include HI layers in minor proportions in order to account for the asymmetric peak shape of ~12 Å diffraction maximum (HI/2W/1W/0W=15/0/85/0 and 3/0/97/0, respectively; Table S2, Fig. S1B). In addition, two additional MLSs were used to account for the increase in intensity of the diffraction maximum located ~13 Å (Fig. S1B). Both contributions are dominated by HI layers in contrasted proportion with minor contribution of 1W layers (HI/1W=88/12 and 56/44, respectively; Table S2, Fig. S1B). The combination of the four MLSs leads to an overall layer content of HI/2W/1W/0W=30/0/70/0. Note that a partial layer ordering (Reichweite parameter =1) was considered for the MLS composed as HI/1W=56/44 (the junction probability between two neighbor 1W layers in the stack referred to as $P_{1W\text{-}1W}$=0.33 is lower than the relative abundance of 1W layers, i.e., 0.44; Table S2, Fig. S1B) in order to reproduce peak shape and intensities for the whole 00ℓ reflection series. Such type of partial ordering of layer was recently reported by Lanson *et al.*[3] on vermiculite also altered in acidic conditions. Due to the broad diffraction bands noticed for the peak at ~13 Å, lower N values were considered (N=15; Table S2) for most of the MLSs considered, except for

the contribution containing high amounts of 1W layers (N=50; Table S2) and likely corresponding to a contribution of unaltered vermiculite. Again two types of 1W layers with contrasted layer-to-layer distances were considered during the modeling exercise, whereas a value of ~14.0 Å was considered for HI layers, in agreement with that reported by Lanson *et al.*[3] (Table S2). The experimental XRD pattern collected after longer alteration time (i.e., 1055h; Fig. S1C), was reproduced using again four MLSs with random interstratification (Table S2). As for the previous sample, a MLS dominated by 1W layers and likely corresponding to pristine vermiculite is present but the three other contributions are mainly composed by HI layers. In agreement with the increase in intensity of the peak at ~14.0 Å as compared to the less altered sample, the three MLSs dominated by HI layers now account for 85% of the diffracted intensity. This leads to a rise of HI layers contribution in the total layer content (HI/2W/1W/0W=56/0/41/3; Table S2).

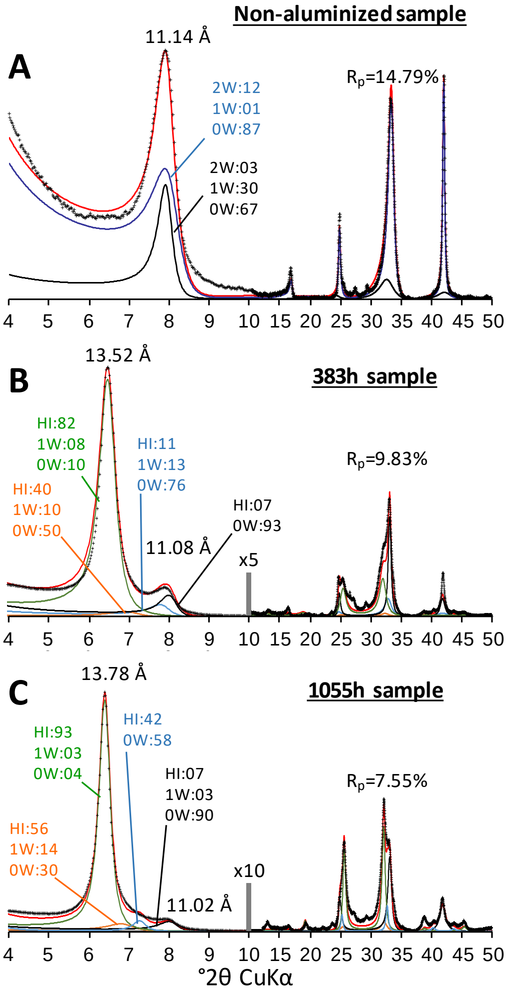

Figure S2. Comparison between the experimental (crosses) and calculated (solid red lines) XRD patterns recorded at 30% relative humidity for Cs^+-saturated material. (A) Reference non-altered Cs^+-vermiculite sample. (B) Sample altered for 383 hours. (C) Sample altered for 105 hours. The other notations and labels are described in Fig. S1.

After Cs^+-saturation of the three samples, the obtained experimental XRD patterns display noticeable changes in both peak position and intensity (Fig. S2). The experimental XRD pattern collected for pristine Cs^+-saturated vermiculite (Fig. S2A) was fitted considering two MLSs. Both MLSs are dominated by 0W layers but contain minor amounts of 2W and 1W layers, leading to an overall layer content of HI/2W/1W/0W=0/10/6/84 (Table S2). In order to correctly reproduce the experimental XRD pattern, the layer-to-layer distance attributed to Cs^+-saturated 0W layers set at 10.76 Å is consistent with the value previously reported by Sawhney[9]. Like for Na^+-saturated specimens, experimental XRD patterns obtained for Cs^+-saturated altered vermiculite samples display two main diffraction maxima (Figs. S2B and C). The first peak near ~13 Å corresponds to MLSs dominated by HI layers whereas the low-intense diffraction maxima located at ~11 Å corresponds to structure containing large proportion of 0W layers. As for their Na^+-saturated counterparts, the two XRD patterns of altered Cs^+-vermiculite samples were fitted assuming four MLSs (Fig. S2B and C). Two MLSs are dominated by HI layers whereas the two others mostly contain 0W layers (Table S2). With increasing alteration time (i.e., from 383 to 1055h), the increase in overall HI layers is accounted for by both (i) the rise of the contribution of HI-rich MLSs to the diffracted intensity and (ii) the increase of relative proportion of HI layers in the different MLSs. The evolution of both aspects is responsible for the decrease in intensity of the diffraction maxima at ~11 Å and for the narrowing of the band at ~13 Å of the experimental patterns (Fig. S2B and C). For sample altered during 1055h, it was necessary to consider an interstratified structure with maximum possible degree of ordering (R1-MPDO; P_{HI-HI}=0.0) for one of the MLS with similar proportion of HI and 0W layers (HI:0W=42:58; Table S2). Finally note that despite the significant differences between experimental XRD patterns collected for Na^+- and Cs^+-saturated samples (Figs. S1 and S2), the relative proportions of HI layers are similar for a given alteration time.

Table S2. Structural parameters used to reproduce experimental X-ray diffraction patterns of the reference Na^+- and Cs^+-vermiculite samples and altered samples for 383 and 1055 hours.

Sample		Na^+-saturated samples			Cs^+-saturated samples		
		0h	383h	1055h	0h	383h	1055h
MLS#1	S cont. (%)[a]	58	37	14	83	41	49
	HI/2W/1W/0W[b]	0/6/94/0	3/0/97/0	95/0/5/0	0/12/1/87	7/0/0/93	93/0/3/4
	CSD size[c]	50	50	17	20	16	14
	Reichweite parameter	R0	R0	R0	R0	R0	R0
MLS#2	S cont. (%)[a]	30	24	16	17	39	35
	HI/2W/1W/0W[b]	0/5/95/0	15/0/85/0	3/0/97/0	0/3/30/67	82/0/8/10	7/0/3/90
	CSD size[c]	50	15	17	20	16	14
	Reichweite parameter	R0	R0	R0	R0	R0	R0
MLS#3	S cont. (%)[a]	12	11	35	-	17	7
	HI/2W/1W/0W[b]	0/0/75/25	88/0/12/0	40/0/50/10	-	11/0/13/76	56/0/14/30
	CSD size[c]	50	15	17	-	16	14
	Reichweite parameter	R0	R0	R0	-	R0	R0
MLS#4	S cont. (%)[a]	-	28	35	-	3	9
	HI/2W/1W/0W[b]	-	56/0/44/0	80/0/20/0	-	50/0/10/40	42/0/0/58
	CSD size[c]	-	15	17	-	16	14
	Reichweite parameter junction probabilities[d]	-	R1 P_{1W-1W}=0.33	R0	-	R0	R1 P_{HI-HI}=0.00
Total layer abundance[e]	HI/2W/1W/0W	0/5/92/3	30/0/70/0	56/0/41/3	0/10/6/84	38/0/6/56	56/0/4/40
Layer-to-layer distances[f]	HI	-	14.00	14.00	-	13.95	13.96
	2W	14.75	-	-	14.90	-	-
	1Wa	11.91	11.97	11.97	11.86	11.95	11.97
	1Wb	12.48	12.50	12.50	-	-	-
	0W	9.80	10.00	10.00	10.76	10.79	10.80

[a] Relative proportions of the different mixed-layer structures (MLS) to the diffracted intensity. [b] Relative proportions of the different layer types in these contributions. HI, 2W, 1W, and 0W stand for hydroxy-interlayered, bihydrated, monohydrated, and dehydrated layers, respectively. [c] Mean size of the coherent scattering domains along the c* axis (in layers). [d] Reichweite parameter (R0=randomly interstratified structure; P_{1W-1W} and P_{HI-HI}: junction probability between two neighboring 1W and HI layers, respectively). [e] The total relative contributions of HI, 2W, 1W, and 0W layer types in the samples. [f] Layer-to-layer distances for HI, 2W, 1W, and 0W layers. 1Wa and 1Wb stand for the two types of 1W layers with contrasted layer-to-layer distances.

REFERENCES

1. Parkhurst, D. L. & Appelo, C. A. J. Description of input and examples for PHREEQC version 3 - A computer program for speciation, batch-reaction, one-dimensional transport and inverse geochemical calculations. (2013).
2. Tertre, E., Ferrage, E., Bihannic, I., Michot, L. J. & Prêt, D. Influence of the ionic strength and solid/solution ratio on Ca(II)-for-Na+ exchange on montmorillonite. Part 2: Understanding the effect of the m/V ratio. Implications for pore water composition and element transport in natural media. *J. Colloid Interface Sci.* **363,** 334–47 (2011).
3. Lanson, B. *et al.* Experimental aluminization of vermiculite interlayers: An X-ray diffraction perspective on crystal chemistry and structural mechanisms. *Geoderma* **249-250,** 28–39 (2015).
4. Ferrage, E., Lanson, B., Sakharov, B. A. & Drits, V. A. Investigation of smectite hydration properties by modeling experimental X-ray diffraction patterns: Part I. Montmorillonite hydration properties. *Am. Mineral.* **90,** 1358–1374 (2005).
5. Ferrage, E., Lanson, B., Michot, L. J. & Robert, J. L. Hydration properties and interlayer organization of water and ions in synthetic na-smectite with tetrahedral layer charge. Part 1. Results from X-ray diffraction profile modeling. *J. Phys. Chem. C* **114,** 4515–4526 (2010).
6. Howard, S. A. & Preston, K. D. Profile fitting of powder diffraction patterns. *Rev. Mineral. Geochemistry* **20,** 217–275 (1989).
7. de la Calle, C. & Suquet, H. in *Hydrous Phyllosilicates (Exclusive of Micas)* (Bailey, S. W.) 455 – 496 (Mineralogical Society of America, 1988).
8. de la Calle, C., Suquet, H., Dubernat, J. & Pezerat, H. Mode d’empilement des feuillets dans les vermiculites hydratées à ‘deux couches’. *Clay Miner.* **13,** 275–297 (1978).
9. Sawhney, B. L. Kinetics of Cesium Sorption by Clay Minerals. *Soil Sci. Soc. Am. Proc.* **30,** 565–569 (1966).
